# Assessing Spatial Representativeness of Global Flux Tower Eddy-Covariance Measurements Using Data from FLUXNET2015

**DOI:** 10.1038/s41597-024-03291-3

**Published:** 2024-06-03

**Authors:** Junjun Fang, Jingchun Fang, Baozhang Chen, Huifang Zhang, Adil Dilawar, Man Guo, Shu’an Liu

**Affiliations:** 1 State Key Laboratory of Resource and Environmental Information System, Institute of Geographic Sciences and Natural Resources Research, Chinese Academy of Sciences, Beijing, China; 2 University of Chinese Academy of Sciences, Beijing, 100049 China; 3 Jiangsu Center for Collaborative Innovation of Geographical Information Resources Development and Application, Nanjing, 210023 China; 4 State Key Laboratory of Earth Surface and Ecological Resources, Faculty of Geographical Science, Beijing Normal University, Beijing, 100875 China; 5 School of Geographical Sciences, Faculty of Science and Engineering, University of Nottingham, Ningbo, 315100 China; 6 School of Remote Sensing and Geomatics Engineering, Nanjing University of Information Science and Technology, Nanjing, 210044 China

**Keywords:** Scientific community, Ecology

## Abstract

Large datasets of carbon dioxide, energy, and water fluxes were measured with the eddy-covariance (EC) technique, such as FLUXNET2015. These datasets are widely used to validate remote-sensing products and benchmark models. One of the major challenges in utilizing EC-flux data is determining the spatial extent to which measurements taken at individual EC towers reflect model-grid or remote sensing pixels. To minimize the potential biases caused by the footprint-to-target area mismatch, it is important to use flux datasets with awareness of the footprint. This study analyze the spatial representativeness of global EC measurements based on the open-source FLUXNET2015 data, using the published flux footprint model (SAFE-f). The calculated annual cumulative footprint climatology (ACFC) was overlaid on land cover and vegetation index maps to create a spatial representativeness dataset of global flux towers. The dataset includes the following components: (1) the ACFC contour (ACFCC) data and areas representing 50%, 60%, 70%, and 80% ACFCC of each site, (2) the proportion of each land cover type weighted by the 80% ACFC (ACFCW), (3) the semivariogram calculated using Normalized Difference Vegetation Index (NDVI) considering the 80% ACFCW, and (4) the sensor location bias (SLB) between the 80% ACFCW and designated areas (e.g. 80% ACFCC and window sizes) proxied by NDVI. Finally, we conducted a comprehensive evaluation of the representativeness of each site from three aspects: (1) the underlying surface cover, (2) the semivariogram, and (3) the SLB between 80% ACFCW and 80% ACFCC, and categorized them into 3 levels. The goal of creating this dataset is to provide data quality guidance for international researchers to effectively utilize the FLUXNET2015 dataset in the future.

## Background & Summary

Due to global land use change and fossil fuel emissions, a large number of greenhouse gases are being released into the atmosphere, resulting in global warming and climate change^1,2^. Carbon dioxide (CO_2_), being one of the primary greenhouse gases, plays a vital role in regulating the earth’s surface temperature through radiative forcing and the greenhouse effect. The cumulative fossil CO_2_ emissions during 1850–2021 were 465 ± 25 GtC^3^. To mitigate the concentration of CO_2_ in the atmosphere, there has been a growing focus on understanding the carbon cycle processes and the carbon sequestration capacity of terrestrial ecosystems^4,5^. For example, Ahlstrom *et al*.^6^ reported that terrestrial ecosystems can substantially sink atmospheric CO_2_ accounting for approximately a quarter of anthropogenic emissions on an annual average. The research investigating the role of terrestrial ecosystems in regulating the absorption and emission of atmospheric CO_2_ is useful for a better understanding of the carbon cycle exchange between terrestrial ecosystems and the atmosphere. It aids in formulating global carbon emissions strategies and coping with global climate change.

The net ecosystem CO_2_ exchange (NEE) shows a small difference between the two large CO_2_ fluxes: photosynthesis (GPP: gross primary production) and ecosystem respiration (ER)^7^. The quantification of NEE indicates whether an ecosystem acts as a net sink or source of atmospheric CO_2_^8^. NEE can be measured by the eddy-covariance (EC) technology, thereby improving our understanding of global change biology^9^. The EC method is reliable for determining the ecosystem-level flux and is widely used to assess ecosystem CO_2_ exchange^10,11^. Consequently, numerous flux networks have been established worldwide, such as ChinaFlux, AmeriFIux, CarboEurope, OzFlux, Fluxnet-Canada, AsiaFlux, and KoFlux. FLUXNET links these regional networks and has experienced rapid growth over the last three decades. Long-term continuous flux observations have immensely enhanced our understanding of carbon, water, and energy cycles in terrestrial ecosystems^12,13^ as well as feedback of ecosystems to global climate change^14^.

EC systems are usually established in ecosystems with low spatial variability of vegetation structural characteristics to reduce measurement uncertainty^15,16^. However, spatial heterogeneity is inevitable in some ecosystems with fragmented landscapes. Potential uncertainties (or representativeness) related to spatial and temporal scales need to be addressed, as this may significantly change the applicability of EC tower data^8,17^.

The flux footprint model is used to describe the spatial extent of the surface area, which contributes to measuring turbulent flux at a specific point, considering specific atmospheric conditions and surface characteristics^18–20^. Several footprint models have been proposed for flux measurements^19,21,22^. Initially, the footprint model was extended to two dimensions (i.e. FSAM) by Schmid^20^, but it was only applicable to neutral and moderately unstable atmospheric stability, with a limited range of crosswind turbulence intensity. Subsequently, an analytical model was developed by Kormann and Meixner to describe the crosswind integrated and distributed footprint under all conditions of atmospheric stability^23^. Kljun *et al*.^18^ presented a simple two-dimensional parameterization for flux footprint prediction (FFP), which is applicable across a wide range of boundary layer conditions and measurement heights. Moreover, it has been applied to produce influential scientific results^24^.

Representativeness describes the extent to which a set of flux measurements taken in a given space-time domain accurately reflect the actual flux conditions in different space-time domains^15,19^. Several studies have examined the representativeness of flux networks at a regional scale, specifically to analyze whether measured data from flux towers reflect the carbon flux in a region^25–28^. Previous studies have also investigated the spatial representativeness of EC networks at the national scale, such as AmericaFlux, and the Canadian Carbon Program^15,19,29^. For example, Chu *et al*.^24^ carried out a representativeness study for all AmeriFlux sites involving a footprint analysis combined with enhanced vegetation index (EVI) and land cover data and evaluated potential biases as a consequence of the footprint-to-target-area mismatches. However, comprehensive global footprint analysis and representative assessment are still lacking.

FLUXNET2015 is the latest globally coordinated EC product, processed and made easily accessible. It has been widely used in different fields because of open access. For example, it has been applied in a wide range of studies, such as calibration of mechanical ecosystem models, and validation of remote sensing-based estimation at different spatial resolutions^30^. However, the FLUXNET2015 network exhibits inherent inhomogeneity in vegetation characteristics^31^. A standard approach such as footprint analysis must be employed to understand the variations in spatial homogeneity and nonuniform sampling patterns. However, the footprint and representativeness of the FLUXNET2015 dataset remain unknown. Therefore, in this study, we used the FLUXNET2015 dataset to analyze the annual cumulative footprint climatology (ACFC) of each flux tower. We calculated the 80% ACFC weighted (ACFCW) land cover percentage designed to measure vegetation functional types (dominant land cover type), the surface semi-variogram considering the footprint weights with vegetation index data, and the sensor location bias (SLB) between the 80% ACFCW NDVI and mean NDVI of designated areas (e.g. 80% ACFC contour (ACFCC) and window sizes) to show the detail information of mismatch of a single flux tower.

**Fig. 1:**
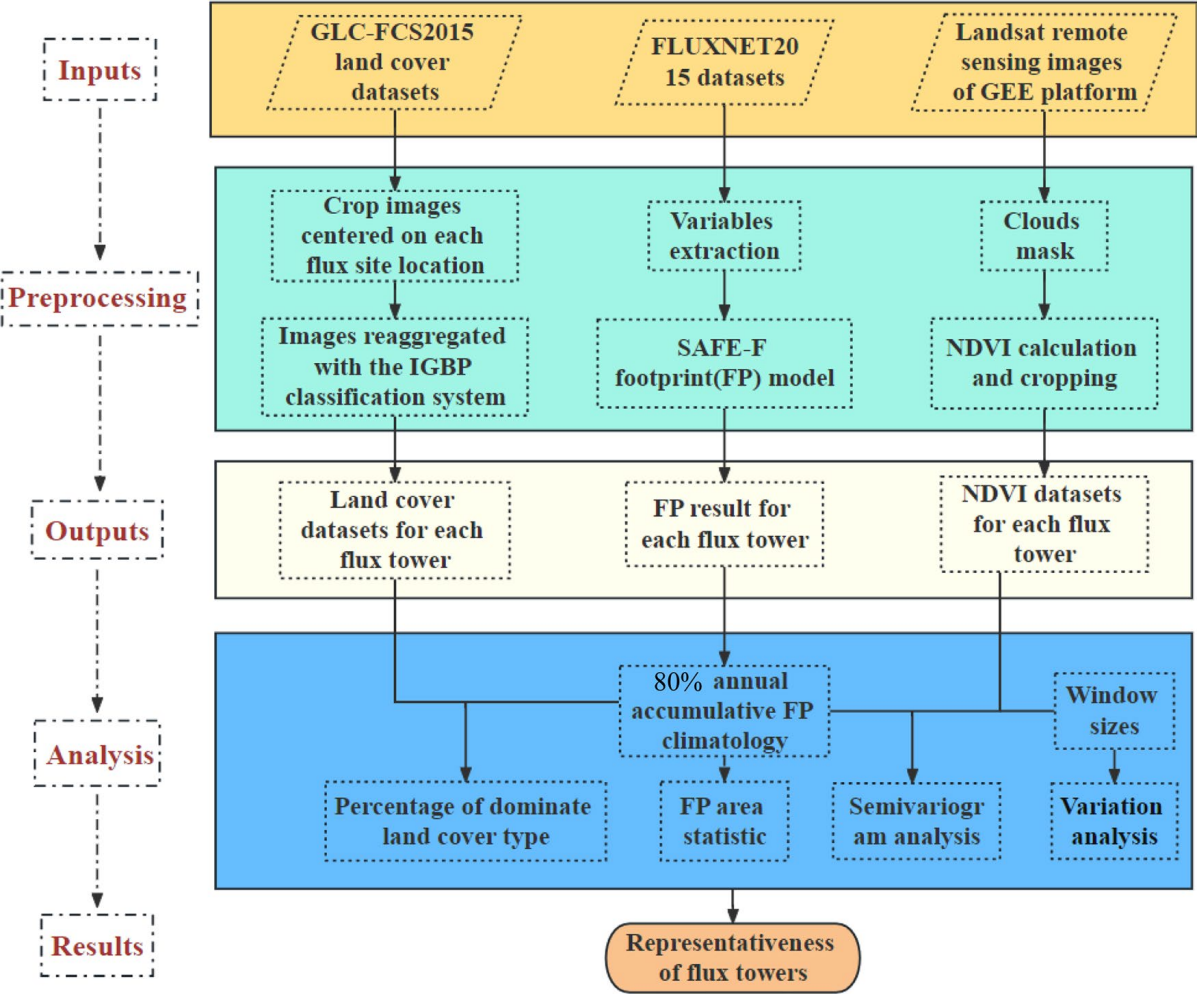
Description of the main framework.

The application of our research follows the two concepts. Firstly, we evaluated the heterogeneity or variation within the 80% ACFC, such as the percentage of the dominant land cover type seen by the tower. It can help users how to interpret the flux data. For example, the results inform users if it contains information from one or multiple land cover types. It can also indicate when benchmarking a site’s data with model simulation (e.g., a single-point run of an Earth system model), which plant functional type should the model prescribe. Secondly, the EC measurement value represents the integral flux over its footprint area, which may not match the scale of the respective model grid or remote sensing pixel. To address these problems, it is crucial to assess the representativeness of the measurements by evaluating the variation between the footprints and designated areas (e.g., fixed cut-off areas). This part of the results provides insights into whether it is appropriate to pair the flux data with other spatially explicit data (e.g., remote-sensing retrievals, reanalysis, or gridded products) that have different spatial extents from the flux footprints. The current findings are valuable for the worldwide user community to enhance the understanding of flux tower data and conduct more accurate research. The systematic framework of the current study is illustrated in Fig. 1.

## Methods

### Selection and description of sites from the FLUXNET2015 dataset

FLUXNET2015 Dataset is publicly available from the community data portal (https://fluxnet.org/)^32^. We selected suitable EC sites and used them to calculate the ACFC. 199 sites were identified out of 212 sites that had all the footprint model required fields that were necessary to drive the SAFE-f. footprint model. The basic information on the selected flux sites can be found here (Table S1, 10.6084/m9.figshare.24884292).

**Fig. 2:**
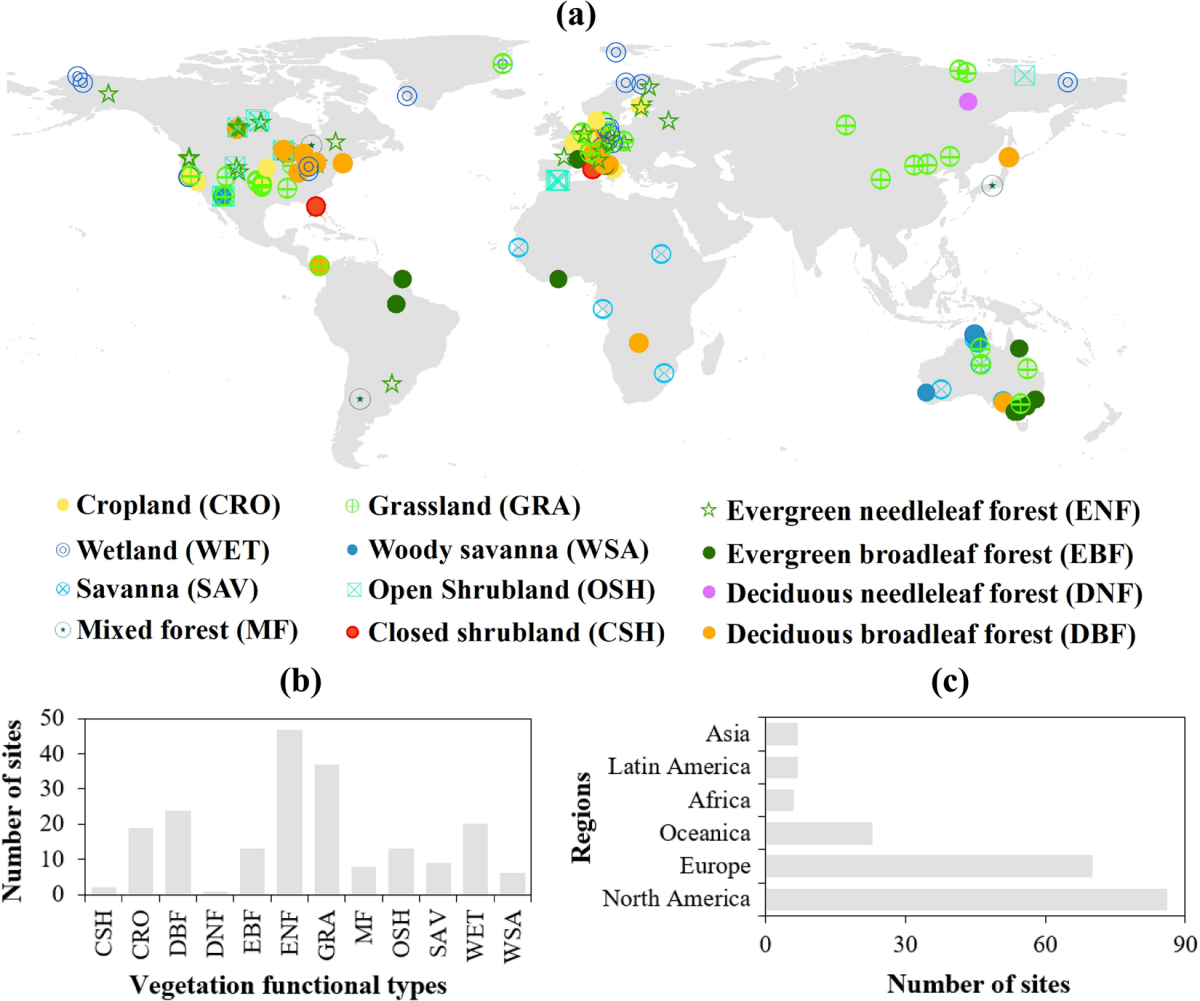
Distribution of elected flux towers from FLUXNET2015 Dataset and their statistic analysis from International Geosphere Biosphere Programme (IGBP) classifications and regional perspective.

Fig. 2 shows the distribution map of the sites and their vegetation function types. It can be seen that most sites are of evergreen needle-leaf forests (ENF) type (with 47 sites), followed by grassland (GRA) type (with 37 sites). The two types of sites account for 42.2% of the total number flux towers. There is only one flux site of the deciduous needle-leaf forest (DNF) type, followed by two sites of the closed shrubland (CSH) type. In addition, other types of sites are between 6–24. Geographically, North America has the largest number of sites (86 sites total), and Europe has the second largest number(70 sites total). 78.4% of the total flux tower sites are in North America and Europe while Africa has only 6 sites.

### Footprint climatologies calculation model

The source areas of the measured vertical fluxes (e.g. net ecosystem exchange) can be estimated with footprint models^18,22,23,33^. Footprint represents the contribution of the source area formed by the corresponding surface flux field upwind at the measurement point to observe CO_2_ flux at sensor height^22,23,34^. In recent years, with the development of the flux footprint function different footprint calculation models have been developed such as the FSAM model^35^, KM01 model^34^, and the Simple Analytical Footprint based on Eulerian coordinates for scalar Flux (SAFE-F) model^19^, and simplified footprint model-flux footprint prediction (FFP)^17^. Each of them has its advantages and disadvantages. For the multi-site FLUXNET2015 dataset, where the measurement height is definitely within the surface layer, we used a widely used and simplified footprint model-SAFE-f model^19^ to compute the footprint of individual flux towers. The SAFE-f model is effective for a wide range of atmospheric conditions^19^. Its versatility makes it suitable for estimating footprints in practical situations, thereby facilitating the footprint calculation of the FLUXNET2015 dataset in our study. To enhance the accuracy of footprint estimates, we conducted comparisons and integrated the footprints generated by SAFE-f with those produced by FFP.

To calculate the footprint of FLUXNET2015, we need input including measurement height (H_m_), surface roughness length (Z_0_), wind speed (WS), wind direction (WD), friction wind velocity (USTAR), the standard deviation of the lateral wind speed (*σ*_*v*_), the planetary boundary layer height (pblh) and Obukhov stability (L). Meteorological fields such as WS, WD, and USTAR for the FFP model are driven from the FLUXNET2015 Dataset, and other input variables such as H_m_ are obtained from the BADM (Biological, Ancillary, Disturbance, and Metadata) data (https://fluxnet.org/). In particular, Z_0_ is approximated as 10% of canopy height (H_c_) here^36^, and we estimated the zero-plane displacement height as 67% of H_c_^37^. As for H_c_, we derived for grassland, wetland, and cropland weekly following the method from Chu *et al*.^38^, for other vegetation function type sites (e.g. forest, shrubland, and savanna), we get the annual H_c_ from the BADM data. The pblh was calculated by methods from Kljun *et al*.^18^. For *σ*_*v*_, FLUXNET2015 did not provide this variable, here we collected from ICOS (https://www.icos-cp.eu/)^39–56^ and AmeriFlux BASE data portals (https://ameriflux.lbl.gov/)^57–75^ for 37 sites (169 site-year) of data (for details, please refer to Table S2, 10.6084/m9.figshare.24884292), and developed a random forest machine learning model to predict the *σ*_*v*_ of the remaining sites. The model was trained and validated using the 169 site-year of *σ*_*v*_ data (N = 1,332,045). The model used 7 predictor variables—USTAR, WS, incoming shortwave radiation, atmospheric pressure, Z_0_, H_m_, and H_c_ which are thought theoretically relevant to cross-wind velocity. It displayed robust performance with 5-fold cross-validation (R^2^ = 0.780, mean absolute error (MAE) = 0.138 m s^−1^). More information on how the model was validated and tested can be found in the supplementary materials.

The flux footprint, f, is defined as the transfer function between sources or sinks of passive scalars at the surface, Q_c_, and the turbulent flux, F_c_, measured at a receptor at height z_m_ mounted above the origin (0,0)^18^ (Eq. 1).

1 $${F}_{c}\left(0,0,{z}_{m}\right)=\mathop{\int }\limits_{\Omega }{Q}_{c}(x,y)f(x,y)dxdy$$

where x indicates the upwind distance, and *Ω* is the integration domain. For simplicity, the vertical reference in f is neglected as the footprint function is always specific to a given measurement height. It follows that the footprint function is proportional to the flux increment arising from a single unit point source or sink, Q_u_ (Eq. 2).

2 $$f(x,y)=\frac{{F}_{c}\left(0,0,{z}_{m}\right)}{{Q}_{u}\left(x,y\right)}$$

where F_c_ is a flux density (per unit area) and Q_u_ is a source or sink integrated over a unit area.

For more details about the procedure, please refer to Kljun *et al*.^18^.

The footprint of each flux tower is calculated using half-hour frequency data, generating a two-dimensional gridded map of footprint weights covering the 80% ACFC. Owing to the FPP model limitation, we filtered the half-hour data according to the conditions in (Eq. 3). Different sites provide different years’ data of FLUXNET2015, considering the time match of footprint with land cover(we use GLC-FCS-2015, described in the next section), we choose data as close as possible to the year 2015 (details can be found in Table S1, 10.6084/m9.figshare.24884292). We extract the drive fields from each tower dataset, tower meta-information, and predicted variable data to drive the FFP model to get the ACFC.

3 $$\left(\begin{array}{c}\frac{{z}_{m}}{L}\ge -15.5\\ 20{z}_{0} < {z}_{m} < 0.8pblh\end{array}\right)$$

To obtain the ACFC of a given flux tower, the (half-) hourly footprint f(x, y) for (x, y) location was accumulated to generate monthly, seasonal then annual values (thus providing ACFC). Because the uncertainty of footprint models increases with upwind distance from the receptor^18^, we truncated the footprint climatologies at the 80% contour of source weights and conducted all subsequent analyses following the methodology suggested by Chen *et al*.^15,19,29^. Their sensitivity tests showed that the 80% contour cutout selection had a marginal influence on the final results.

### Land surface cover pre-processing and analysis

In this study, we used Global Land Cover with Fine Classification System at 30 m in 2015 product (GLC_FCS30-2015) as the land surface characteristic to analyze the representativeness of flux towers. GLC_FCS30-2015 was produced by a multi-temporal classification method based on the random forest using a prior spectral library which builds by combining the time-series MCD43A4 NBAR (Nadir bidirectional reflectance distribution function adjusted reflectance) surface reflectance products and CCI_LC landcover products^76^. The spatial resolution is 30 m × 30 m, the simulated map contains approximately 30 landcover types and the reported accuracy achieved an overall of 82.5%.

To match the classification of GLC_FCS30-2015 products with the International Geosphere Biosphere Programme (IGBP) classification of flux towers, we merged and consolidated the GLC_FCS30-2015 land cover types into 18 groups as same as the IGBP classification system (Table 1). With each tower location as the center, we extracted land cover products within ± 0.02 degrees of longitude and latitude direction to overlaid with the ACFC. Then we calculated the 80% ACFCW percentage of each land cover type.

**Table 1:** Land cover classes of GLC_FCS30-2015 and aggregation schemes to International Geosphere Biosphere Programme (IGBP) classification system.

GLC_FCS30-2015 ID	Classification System of GLC_FCS30-2015	IGBP ID	IGBP land cover types
10	Rainfed cropland	12	Croplands
11	Herbaceous cover	12	Croplands
12	Tree or shrub cover (Orchard)	12	Croplands
20	Irrigated cropland	12	Croplands
50	Evergreen broadleaved forest	2	Evergreen Broadleaf Forest
60	Deciduous broadleaved forest	4	Deciduous Broadleaf Forest
61	Open deciduous broadleaved forest (0.15 < fc < 0.4)	4	Deciduous Broadleaf Forest
62	Closed deciduous broadleaved forest (fc > 0.4)	4	Deciduous Broadleaf Forest
70	Evergreen needle-leaved forest	1	Evergreen Needleleaf Forest
71	Open evergreen needle-leaved forest (0.15 < fc < 0.4)	1	Evergreen Needleleaf Forest
72	Closed evergreen needle-leaved forest (fc > 0.4)	1	Evergreen Needleleaf Forest
80	Deciduous needle-leaved forest	3	Deciduous Needleleaf Forest
81	Open deciduous needle-leaved forest (0.15 < fc < 0.4)	3	Deciduous Needleleaf Forest
82	Closed deciduous needle-leaved forest (fc > 0.4)	3	Deciduous Needleleaf Forest
90	Mixed leaf forest (broadleaved and needle-leaved)	5	Mixed Forest
120	Shrubland	7	Open Shrublands
121	Evergreen shrubland	7	Open Shrublands
122	Deciduous shrubland	7	Open Shrublands
130	Grassland	10	Grasslands
140	Lichens and mosses	16	Barren or Sparsely Vegetated
150	Sparse vegetation (fc < 0.15)	16	Barren or Sparsely Vegetated
152	Sparse shrubland (fc < 0.15)	16	Barren or Sparsely Vegetated
153	Sparse herbaceous (fc < 0.15)	16	Barren or Sparsely Vegetated
180	Wetlands	11	Permanent Wetlands
190	Impervious	13	Urban and Built-Up
200	Bare areas	16	Barren or Sparsely Vegetated
201	Consolidated bare areas	16	Barren or Sparsely Vegetated
202	Unconsolidated bare areas	16	Barren or Sparsely Vegetated
210	Waterbody	18	Water Bodies
220	Permanent ice and snow	15	Permanent Snow and Ice
250	Filled value	17	Unclassified

Table 1 shows that for woody savannas, savannas, and closed shrublands, there is no GLC_FCS30-2015 land cover type to match. For the mentioned land cover-type flux towers, we will correct directly their percentage value. For example, after preliminary aggregation tests, we found that deciduous broadleaved forest (DBF) is the dominant land cover type for woody savannas (WSA) sites, evergreen needle-leaved forest (ENF) is the dominant land cover type for savannas (SAV) sites, and DBF is the dominant land cover type for closed shrubland (CSH) sites. Based on aggregation tests, the percentage value of DBF to WSA, ENF to SAV and DBF to CSH was corrected for concerning sites in our study. During this procedure, images from Google Maps were used as a reference.

### Vegetation index and analysis

The NDVI is used to describe the land surface heterogeneity in this study. It shows vegetation canopy density and green leaf biomass and is computed using the following equation^77^ (Eq. 4). The Landsat images were used to calculate the NDVI during the year corresponding to EC flux data from the Google Earth Engine platform (https://earthengine.google.com/). We extracted a range of ±0.02 degrees centered on the location of each flux tower. We selected to extract NDVI data from the Landsat images with less than 5% cloud cover to reduce the influence of cloud cover contamination. Annual mean composite NDVI images were formed and used for semivariogram and window size analysis corresponding to the ACFC. To ensure a sufficient sample size for semivariogram analysis, we exported spatial resolution data from Google Earth Engine at a resolution of 10 m × 10 m in our research.

4 $$NDVI=\left({b}_{nir}-{b}_{red}\right)/\left({b}_{nir}+{b}_{red}\right)$$

Where b_nir_ is the NIR band and *b*_*red*_ is the red band for the Landsat series of sensors, respectively.

### Semivariogram analysis

Semivariogram is a geostatistical method, which is widely used in the study of surface heterogeneity in geosciences^78–81^. It gives a concise and fair description of the scale and pattern of spatial variability. Moreover, it can be applied to regional spatial variability and autocorrelation under the premise of the stationarity assumption^82^. The current study adopted this method to assess the spatial variation (heterogeneity intensity) of underlying vegetation of the flux tower within the  80% ACFC domain using the NDVI.

**Fig. 3:**
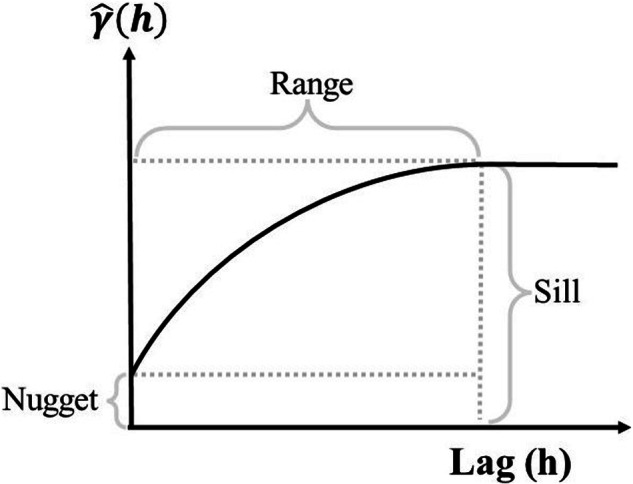
Schematic diagram of the structure and parameters of the semivariance.

A semivariogram describes the dissimilarities between every data pair at a specific distance (h). The average dissimilarity between data separated by “h” is measured by the experimental semivariogram, which is computed as half of the average squared difference between the components of every data pair. The mathematical expression of semivariogram can be defined as following^78^ (Eq. 5)

5 $$\widehat{\gamma }\left(h\right)=\frac{1}{2N(h)}\mathop{\sum }\limits_{\alpha =1}^{N(h)}{\left[z({x}_{\alpha })-z({x}_{\alpha }+h)\right]}^{2}$$

where, $\widehat{\gamma }\left(h\right)$ is an estimate of the semivariance, *N*(*h*) is the number of data pairs for a given distance, *z*(*x*_*α*_), and *z*(*x*_*α*_ + *h*) are data values at h distance. We use this equation to calculate four directions (East-West, North-South, Northeast-Southwest, Northwest-Southeast) semivariogram using 80% ACFCW NDVI values, then we average four directional values to estimate the omnidirectional semivariogram. The spherical model was used to fit the calculated semivariance and estimate the parameters of the model. The parameters of the semivariogram including lag, nugget, sill, and range, which are used to characterize the spatial variation properties are illustrated in Fig. 3. The distance to reach the asymptotic value of the semivariance is called the ‘range’ and indicates the degree of heterogeneity. The plateau of the semivariance estimate in the range is the ‘sill’, which is a measure of the absolute amount of heterogeneity. The larger the semivariance, the less similar the pixels are. The non-zero intercept of the semivariance curve with the Y-axis is the ‘nugget’, which indicates the non-spatial variation due to sampling error.

### Sensor location bias analysis

Sensor location bias (SLB) is commonly employed to assess whether point-to-area representativeness in a specific case is achieved or not^19,29^. In our study, we utilized this metric to quantitatively evaluate the representativeness of each flux site. We followed the equation proposed by Schmid *et al*.^83^, represented as follows (Eq. 6).

6 $$SLB=\frac{NDV{I}_{fp}-NDV{I}_{target}}{NDV{I}_{target}}$$

7 $$NDV{I}_{fp}={\sum }_{j=1}^{J}\left({\varphi }_{j}\times NDV{I}_{j}\right)$$

Where j is an individual pixel, J denotes the total number of pixels within the area of interest (e.g. 80% ACFCC), *φ*_*j*_ and *NDVI*_*j*_ denote the weights of footprint and NDVI value at pixel j, respectively. *NDVI*_*target*_ is the mean NDVI of the targe-area. Here, we calculated the SLB between the 80% ACFCW and 80% ACFCC to effectively represent the representativeness of each flux tower site. In this context, the target area is defined as the region enclosed by the 80% ACFCC.

### Window size analysis

We employed a straightforward window size method to evaluate the representativeness of flux footprints for specific areas based on NDVI. This method offers users insights into the appropriateness of pairing flux data with other spatially explicit data. We also use SLB as a metric to describe the appropriateness. The window sizes (*l*) were chosen following (Eq. 8) in our study.

8 $$l=30\times \left(2\times i-1\right)$$

Where, i = 1,2…,50. For *NDVI*_*fp*_, we selected the 80% ACFCW NDVI value.

### Evaluation of footprint representativeness

Footprint representativeness was evaluated in our research using the following data items: (1) the 80% ACFCW percentage of each land cover type of individual sites, (2) the 45° intervals and omnidirectional semivariogram value calculated based on 80% ACFCW NDVI for each site, and (3) the SLB proxied by NDVI between the 80% ACFCW and designated areas (80% ACFCC).

The first concept is to evaluate the heterogeneity within the 80% ACFC using the percentage of land cover types seen by the tower, and the semivariogram at different directions from data items (1) and (2). It provides significant information to users on how to interpret the flux data. For example, findings can explain which direction the variation was more severe, and which land cover was responsible for it. Moreover, when benchmarking a site’s data with model simulation, results can indicate what plant functional type should the model prescribe. The second concept is to assess the representativeness of 80% ACFC for specific areas surrounding the towers from data item (3), e.g., by examining the variation of NDVI between the footprints and fixed cut-off areas (80% ACFCC). we also provide SLB of different window sizes additionally, these results can inform users whether it is appropriate to pair the flux data with other spatially explicit data (e.g., remote-sensing retrievals, reanalysis, or gridded products) with different spatial extents from the flux footprints.

Finally, all sites were classified into 3 classes in terms of the percentage of dominant land cover type according to Chu *et al*. ‘s criteria^24^. For the omnidirectional semivariogram, we calculated the ratio of the nugget to sill and classified it according to the criteria from Cambardella *et al*.^84^. For SLB between the 80% ACFCW NDVI and mean NDVI within the 80% ACFCC, we calculated the absolute value of SLB (|SLB|) and lastly classified into 3 classes for all flux towers according to Chen *et al*.^29^ (refer to Table 2 for classification details).

**Table 2:** The classification criteria and their level values in three data items (80% ACFCW percentage of dominant land cover type, the omnidirectional semivariogram calculated by 80% ACFCW NDVI, and the sensor location bias between 80% ACFCW and designated areas).

Percentage of dominant land cover type		The omnidirectional semivariation (nugget/sill,%)		Sensor location bias (|SBL|)	
Classification criteria	Levels	Classification criteria	Levels	Classification criteria	Levels
<50	3 (Low)	<25	1 (High)	<0.05	1 (High)
50–80	2 (Medium)	25–75	2 (Medium)	0.05–0.1	2 (Medium)
>80	1 (High)	>75	3 (Low)	>0.1	3 (Low)

## Data Records

The dataset is stored at (10.6084/m9.figshare.24884292)^85^. A detailed description of the dataset of current work is given in Table 3. The generated dataset provides the original data and insightful information for the researcher community to see the area of ACFCC, the 80% ACFCW percentage of each land cover type, and the heterogeneity situation represented by semivariation. Besides, flux towers are classified into 3 classes showing their representativeness levels.

**Table 3:** Data items included in the dataset in this study.

Items ID	Items name	Data format	Descriptions
1	The annual cumulative footprint climatology contour (ACFCC) data	.xls (The original data);.jpg (The figure of original data)	The 50%, 60%, 70%, and 80% ACFCC origin data are circled by points with x and y (unit: meter), which denotes the distance from the sensor location in.xls format calculated by Flux Footprint Prediction (FFP) model. The figures in.jpg format of 50%, 60%, 70%, and 80% ACFCC.
2	Statistical area of ACFCC	.xls	The statistical area of 50%,60%,70%, and 80% ACFCC in m^2^ and km^2^.
3	ACFCC-Landcover overlay map	.jpg	Overlay map of 50%, 60%, 70%, and 80% ACFCC and land cover data.
4	Percentage of land cover types	.xls	The percentage of each land cover category weighted by the 80% ACFC (ACFCW).
5	Semivariogram of NDVI	.xls	The East-West, North-South, Northeast-Southwest, Northwest-Southeast, and omnidirectional semivariogram using 80% ACFCW NDVI. We provided 3 indices of the semivariogram including the range, nugget, and sill. We also provided the omnidirectional nugget-to-sill ratio.
6	Sensor location bias (SLB) analysis result	.xls	We first provided the average NDVI value of the target area (80% ACFCC and area defined by window sizes), and 80% ACFCW NDVI. Then, we calculated the SLB using NDVI between them. Lastly, the absolute value of SLB (|SLB|) was calculated and provided.
7	Representative classification	.xls	We classified flux towers into 3 classes of representativeness according to the percentage of dominant land cover type, omnidirectional semivariation, and SLB result between 80% ACFCW NDVI and mean NDVI within 80% ACFCC.

### Data example

**Fig. 4:**
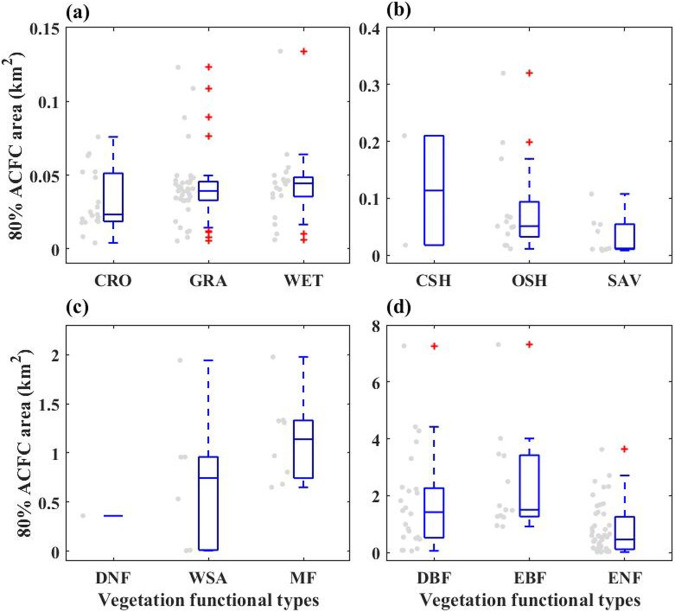
Area of 80% ACFCC divided by vegetation function types. The grey points are the areas of each flux tower distribution, and the blue line within the boxes are the median areas of each type. See Fig. 2 for the full name corresponding to the abbreviation of each vegetation type.

Fig. 4 shows the area of 80% ACFCC data items, which are divided into 12 categories according to the vegetation function types of the flux tower. Also, 50%, 60%, and 70% can be found in item ID 2. From the figure, it can be seen that the flux sites of DBF and EBF vegetation functional type (Fig. 4d) have the largest average footprint area, and the flux sites of grass and crop functional type (Fig. 4a) have the smallest average footprint area. The footprint area ranges from 4030.19 m^2^ to 7.31 km^2^.

**Fig. 5:**
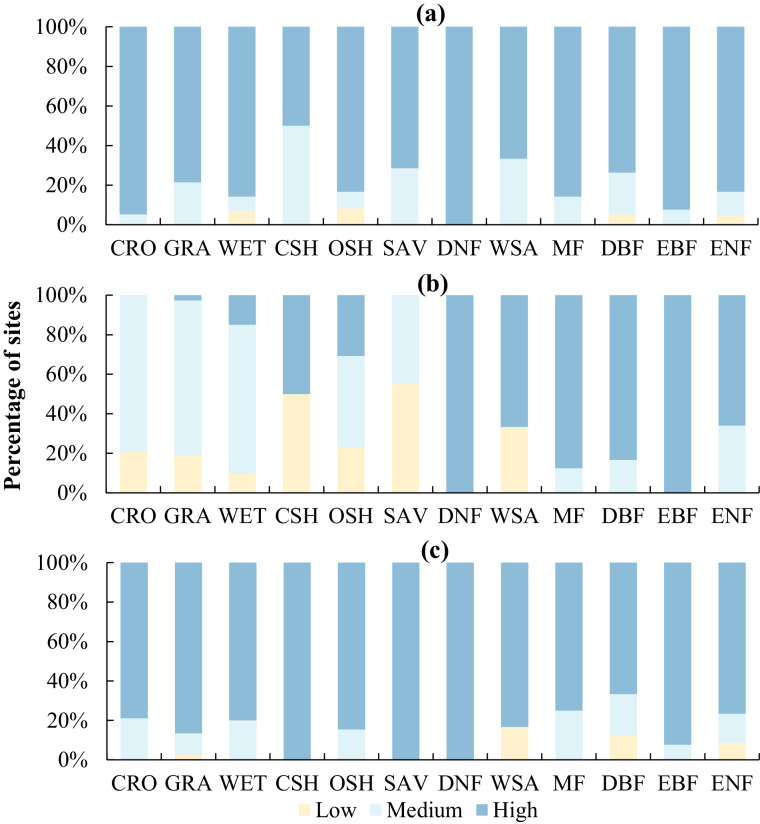
Percentage of sites corresponding to representative levels according to dominant land cover type (**a**), omnidirectional semivariation (**b**), and sensor location bias (**c**) for each vegetation function type. See Fig. 2 for the full name corresponding to the abbreviation of each vegetation type.

**Fig. 6:**
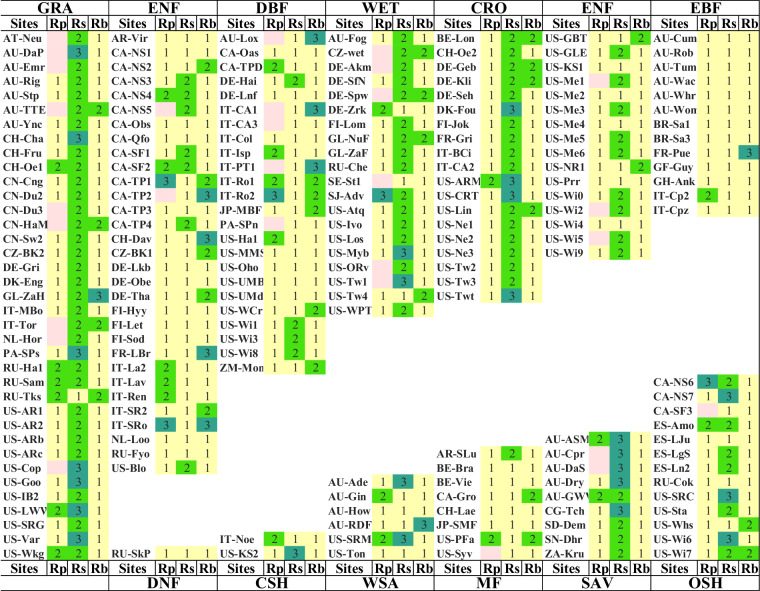
Representativeness levels of flux towers. The first column of each site (Rp) shows the classification by the percentage of dominant vegetation functional types, the second column (Rs) shows the levels by omnidirectional semivariation, and the third column (Rb) is classified by the sensor location bias analysis results. The pink color indicates a mismatch between the dominant vegetation function type of the GLC_FCS30-2015 land cover product and the IGBP at the site.

Fig. 5 is derived from item ID 4, item ID 5, and item ID 6. After analysis, we found that 29 flux sites had mismatches between the dominant land cover type from GLC_FCS30-2015 and the site’s IGBP classification. Therefore, we only focus on the remaining 170 flux sites about this data item (item ID 4, see Fig. 6 for details about the mismatch sites). We divided all sites into 12 categories according to vegetation function types of the flux tower, and the percentage of sites of these three data sets for each vegetation function type is calculated.

Fig. 6 derived from item ID 7, denotes the level of representativeness of flux towers according to the percentage of dominant land cover type, omnidirectional semivariation, and the sensor location bias. We divided all sites into 12 categories according to the vegetation function type of flux sites. There are 3 levels of representativeness: High(1), medium(2), and low(3), the criteria of their levels can be found in Table 2.

## Technical Validation

### The uncertainty in annual cumulative footprint climatology

Biases in the footprint climatology estimates will lead to uncertainty of representativeness for individual flux towers. Several studies have attempted to validate footprint models through field experiments and trace gas release^86–88^. However it is not the scope of this study, we will describe the uncertainty in ACFC in the following aspects.

First, Kljun *et al*.^18^ developed the FFP model and analyzed the sensitivity of the input parameters to the model carefully, they also pointed out the limitation of this model and compared the footprint result of this model to the other three models (HKC00, KM01, and KRC04). More detailed information about the uncertainties of the FFP model can be found in their research.

Second, the FFP has its assumptions, like steady-state conditions, horizontally homogeneous turbulent fields, and no vertical advection. In our research, we are mainly focused on the annual scale footprint which is generated from several (half-) hourly footprints, we think that the instability condition with multiple data only leads to a minimal effect on our final results. Cautions will be required when referring to horizontally homogeneous assumptions. Fluxnet is a network of flux towers that have different underlying surfaces, although EC systems are usually established in ecosystems with low spatial variability of vegetation structural characteristics^16^, spatial heterogeneity is inevitable in some ecosystems with fragmented landscapes. Similar to previous studies^15,24^, our representativeness of footprint is inherently influenced by landscape heterogeneity. For vertical advection, Chu *et al*. highlighted the potential complications introduced by vertical advection in footprint calculations^24^, and suggested using caution when interpreting results from sites influenced by vertical advection, emphasizing the need for thorough, site-specific footprint analyses in such cases.

**Fig. 7:**
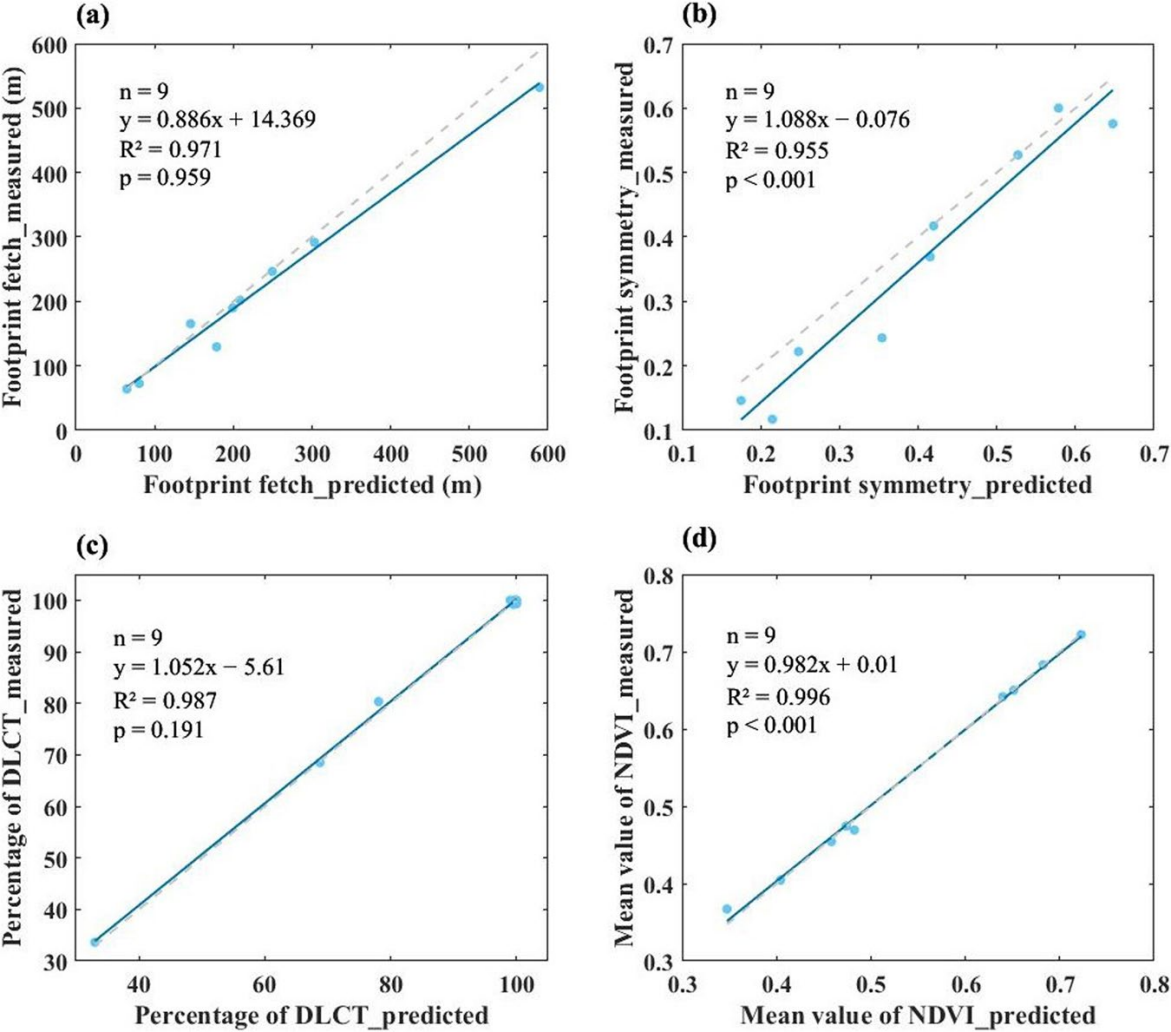
Uncertainty assessment of results by the standard deviation of the lateral wind speed (*σ*_*v*_). (**a**) is the 80% ACFCC fetch calculated using predicted *σ*_*v*_ and measured *σ*_*v*_, (**b**) is the 80% ACFCC symmetry index, (**c**) is the percentage of 80% ACFCW dominant land cover type (DLCT), (**d**) is the mean NDVI value within 80% ACFCC. The used sites can be found in Table S2, the blue line is the fitted line to the blue scatter point, and the grey dotted line is the 1:1 reference line.

Third, the parameters we used in the FFP model will cause uncertainties in the following aspects. For example, the surface roughness length (Z_0_), the zero-plane displacement height, the canopy height (H_c_) estimated for grassland, wetland, and cropland, and the predicted standard deviation of the lateral wind speed (*σ*_*v*_). All these parameters may not be true values of flux sites, thus leading to uncertainties of ACFC for flux towers and the final analysis results. Nonetheless, Chu *et al*.^24^ contend that the impacts on the ultimate evaluation results should be marginal, as analyses were conducted using footprint climatologies, aggregating footprints from many time steps and diverse wind directions. The aggregation process is inclined to mitigate random disparities in the lateral dimensions of half-hourly footprints. We also calculated the ACFC using measured *σ*_*v*_ and predicted *σ*_*v*_ at 9 sites (Table S2) that have data in the same year to analyze the uncertainty introduced by σ_v_ (Fig. 7). We use footprint fetch (maximal distance from the tower location to the 80% ACFCC) and symmetry index (Eq. 9) to characterize the shape of footprint. We also compared the 80% ACFCW dominant land cover type and mean NDVI within 80% ACFCC using measured *σ*_*v*_ and predicted *σ*_*v*_ at these flux sites. All these 4 variables have a good agreement in the footprint calculated by the predicted and measured *σ*_*v*_ (R^2^ > 0.950). So we conclude that using the predicted σ_v_ has a negligible effect on our final results. More information about uncertainty in the *σ*_*v*_ propagated to the ACFC can be found in the supplementary materials.

9 $$SI=\frac{A}{\pi \times fetc{h}^{2}}$$

Where SI is the symmetry index of 80% ACFCC, and A is the area of 80% ACFCC.

Last, in addition to the uncertainty of the model, it should be noted that the representativeness of sites with e.g. a mix of deciduous and evergreen forests could vary greatly in summer and winter. Furthermore, our study only focused on the annual footprint climatology analysis of flux sites, like short-vegetation sites, which have different footprint climatology in different seasons due to changes in canopy height. Therefore, it is recommended to take specific situations into account when using this dataset. We, in this study, only calculated the footprint climatology of flux sites for one year, and did not perform the calculation and variation analysis of the footprint climatology for a long period (e.g., multiple years), which may vary considerably from year to year^26^, so it is recommended that users pay attention to the year in which we calculated when using the data. In the future, multi-year footprints should be calculated to fully understand the inter-annual variability of the data.

### Uncertainty of land cover classification and vegetation index

Errors in land cover type will lead to uncertainty in the assessment of the spatial representativeness of EC flux towers. The land cover data used in the current study have uncertainties in the following aspects that lead to biases in current data analysis. Firstly, the accuracy of the land cover data, as the accuracy of GLC_FCS30-2015 is 82.5%, may affect the current analysis of the percentage of dominant land cover type. Secondly, the difference in the classification system between GLC_FCS30-2015 and IGBP, and the aggregate process may introduce errors for the dominant land cover type statistics. For example, the lichens and mosses type is not so implicit to aggregate by the IGBP classification system. It was aggregated to barren or sparsely vegetated according to the comparison of its classification system with other classification systems^76^, but some sites of them should be aggregated to grassland. To reduce errors in this aggregation process, google earth images (https://earth.google.com/web/) were used as a reference to correct the aggregation process to get a more correct or realistic land cover product. After that, there are 29 mismatches between the site’s IGBP classifications and the dominant land cover obtained from the landcover data products (GLC_FCS30-2015). The same problem can be found in other studies too^24^. These inconsistency highlights require better assessments and improvements of the available landcover data products for the FLUXNET2015 dataset. Lastly, the land cover data are from 2015, while some of the EC flux data are from older years, which may result in potential biases. The present study evaluated the overall sites and found that most of these flux towers were selected at sites with slower land cover changes, over 90% of the sites had no significant land cover changes within 80% ACFC. However, there are a small number of sites that have experienced significant land cover changes, such as US-ORv, ES-Amo, and IT-Isp, due to significant changes in impervious surfaces within their 80% ACFC. If this change in land cover resulted in a mismatch in the dominant vegetation functional type at the flux site, we excluded the corresponding flux site from our analysis (e.g. US-ORv).

Using NDVI as a surrogate for semi-variance and SLB analysis is only a simple estimation because of its limitations. For example, under saturated conditions, it is insensitive to changes in green biomass and vegetation function. In addition, given large seasonal variations of carbon and water fluxes and the single composite NDVI of all the images taken over a whole year was used at each site, the uncertainty in the land surface spatial characterization is worth considering. Although the revisit cycle of Landsat is 16 days, some of the images are contaminated by clouds or the remote sensing images themselves are of very poor quality. Moreover, our study only focuses on one year of footprint analysis, resulting in the lack of NDVI data corresponding to each season for flux stations. We might do a multi-year seasonal or monthly scale footprint climatology analysis in the future. Kim *et al*.^89^ performed a footprint analysis based on single remote sensing image-driven NDVI by assuming that seasonal variation in NDVI proportionally affects all regions within a 6*6 km range and similar phenological development for a single site. Chen *et al*.^15^ also pointed out that when the analysis focused on the annual relative variation of NDVI, it was reasonable to assume that the observed relative variation of NDVI did not change throughout the year. In the above case, although there are uncertainties in using only a single composite NDVI in our study, we think it is still valid.

Another crucial consideration involves the calculation of SLB by averaging NDVI data with different window sizes. In our research, we selected window sizes ranging from 30 m to 2970 m, resulting in areas ranging from 900 m² to approximately 9 km². However, the minimum and maximum areas of 80% ACFC are 4030.19 m² and 7.31 km², respectively. Therefore, for certain flux sites (e.g., grass sites), it may be inappropriate to directly compare the variation between the 80% ACFC and the target areas defined by larger window sizes. In such cases, caution should be exercised because even though the |SLB| may appear small due to the averaging NDVI we use within the target regions, the NDVI spatial difference between the 80% ACFC and the target area could be substantial. To mitigate this situation, we recommend users utilize the SLB data item combined with the corresponding land cover information we provided. This approach will enable more accurate interpretation and analysis, taking into account the specific characteristics of different land cover types.

## Usage Notes

The generated datasets are available from 10.6084/m9.figshare.24884292^85^. The main data file is separated into 7 folders as shown in Table 3. We provided an example of a site here to help users work with any site in the dataset. Because there is only one site of type DNF, we use this site (site_ID: RU-SkP) to describe each data item of the dataset in detail.

### Data item 1

**Fig. 8:**
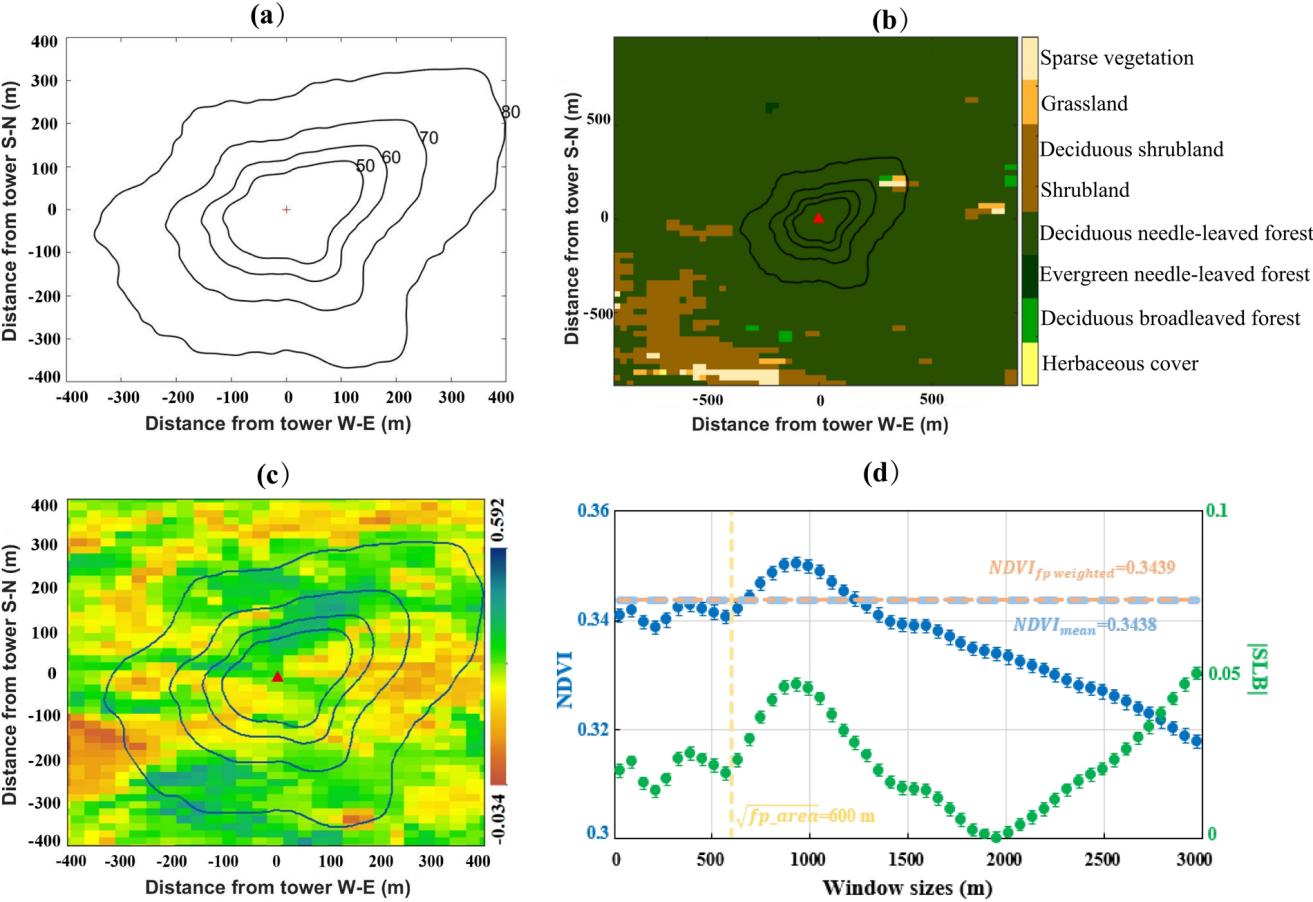
The result provided in the current dataset. (**a**) The ACFCC distribution of RU-SkP flux sites. The contours from inner to outer are 50%, 60%, 70%, and 80%, respectively. (**b**) The ACFCC line overlaid on 30 m spatial resolution land cover data (GLC_FCS30-2015). The red triangle is the site location. (**c**) The ACFCC line overlaid on NDVI derived from Landsat. (**d**) The average NDVI of different window sizes (blue dots), the 80% ACFCW NDVI (the light pink dashed line), the mean NDVI within 80% ACFCC (the light blue dashed line), the window size corresponds to the area of 80% ACFCC, and the absolute value of sensor location bias |LSB| at different window sizes (green dots), respectively.

In our dataset^85^, you can find ‘RU-SkP.xls’ and ‘RU-SkP.jpg’ in the first archive named ‘The annual cumulative footprint climatology contour (ACFCC)’. ‘RU-SkP.xls’ is the ACFCC origin data of this flux tower in ‘.xls’ format. There are four contours including 50%,60%,70%, and 80% of ACFCC. The data columns ‘fp50_x’ and ‘fp50_y’ represent the x and y coordinates of the points on the 50% ACFCC (with the flux site as the origin) in meters. ‘RU-SkP.jpg’ is the figure file of ACFCC, and the red cross symbol is the site location (Fig. 8). The plotting codes we provided here (https://github.com/CodesRes/Representativeness-of-flux-tower-in-FLUXNET2015).

### Data item 2

The second item is named ‘Statistical area of ACFCC’, which includes areas of 50%, 60%, 70%, and 80% of ACFCC. For the RU-SkP flux tower, the areas are 0.04 km^2^, 0.07 km^2^, 0.15 km^2^, and 0.36 km^2^, respectively.

### Data item 3

The third archive is the ‘ACFCC-Landcover overlay map’, ‘RU-SkP.jpg’ is produced by overlay 50%, 60%, 70%, and 80% ACFCC to land cover data (GLC_FCS30-2015)^76^. The color of the legend for each type of land cover that we use in Fig. 8b is the same as the GLC_FCS30-2015 product data used in their study.

### Data item 4

The fourth item is called ‘80% ACFCW percentage of land cover’. We statistic the percentage of each land cover type with 80% AFFCW using GLC_FCS30-2015 data at the RU-SkP site, but the land cover types are aggregated into 18 classes according to the IGBP classification system as Table 1. Finally, the statistical results for this site are shown in Table 4.

**Table 4:** The 80% ACFCW percentage of each land cover type at RU-SkP flux site using GLC_FCS30-2015 data, land covet types are aggregated according to the IGBP classification system.

Land cover types	Deciduous Needleleaf Forest	Barren or Sparsely Vegetation	Deciduous Broadleaf Forest	Open Shrublands	Grasslands
**Percentage**	99.621	0.162	0.096	0.059	0.062

Combining Fig. 8b and Table 4 shows the dominant land cover type is deciduous needle leaf forest (accounting for 99.621%), which is consistent with the vegetation function type of this site. There are 0.162% sparsely vegetation and 0.096 deciduous broadleaf forest approximately on the northeast side of the site within 80% ACFCC. Therefore, when we use EC measurements of this site, we should consider their interference effects. Moreover, when benchmarking this site data with model simulation, the deciduous needle leaf forest should be chosen as the plant functional type for a model prescription.

### Data item 5

The fifth item is the ‘Semivariogram of 80% ACFCW NDVI’. There are five semivariogram values in different directions for the RU-SkP site calculated by 80% ACFCW NDVI (Table 5). In the North-South direction, the semi-variant value was the biggest (0.00124), while in the Ease-West direction, the semivariogram value was the smallest (0.00071). As can be seen from Fig. 8c, there are high NDVI value areas in the North-South direction. However, NDVI values in the East-West direction are relatively low within the 80% ACFCC. This makes the heterogeneity in the North-South direction bigger than in other directions.

**Table 5:** The semivariogram values in different directions for the RU-SkP site were calculated by 80% ACFCW NDVI.

**Site_ID**	**Range (m)**	**Direction**	East-West	Northeast-Southwest	North-South	Northwest-Southeast	Omnidirectional	**Omnidirectional Nugget/Sill**
		**Nugget**	0.00006	0.00022	0.00017	0.00023	0.00017	
RU-SkP	240	**Sill**	0.00071	0.00101	0.00124	0.00117	0.00104	0.165

### Data item 6

The sixth item is ‘Sensor location bias analysis result’. We calculated the mean NDVI within 80% ACFCC and 80% ACFCW NDVI, and the SLB between them. We also provided the mean NDVI value in target areas defined by different window sizes, and the SLB between 80% ACFCW and target areas. For the RU-SkP site, the NDVI and SLB result is shown in Fig. 8d. The maximum mean NDVI is 0.3504 at a 930 m window size and the minimum NDVI is 0.3178 at a 2970 m window size. The mean NDVI in the 80% ACFCC is 0.3438, the 80% ACFCW NDVI is 0.3439, and the SLB between them is 0.05%. According to Table 2, this site is a good representation of the area corresponding to the 80% ACFCC. The SLB between 80% ACFCW NDVI and target areas defined by all window sizes is consistently smaller than 5%, indicating high representativeness levels as per Table 2. As previously mentioned, to ensure an accurate interpretation, this data should be combined with a land cover map. For instance, Fig. 8b illustrates that the maximum window size with which this flux site can be paired is 1000 m. This determination is based on the presence of shrubland influence located 500 m southwest of the site.

### Data item 7

The last item is ‘Representative classification’. We give the percentage of dominant land cover type, the omnidirectional semivariogram, and the |SLB| calculated by NDVI between 80% ACFCW and 80% ACFCC. According to Table 2, we classified the three data into 3 levels which can contribute to users about the representativeness result of these flux sites.

### Supplementary information

Supplementary file for the standard deviation of the lateral wind speed prediction and uncertainties.

## Data Availability

Matlab code for analysis of annual cumulative footprint climatology, land cover type, semivariogram, window size, and for plotting are provided at https://github.com/CodesRes/Representativeness-of-flux-tower-in-FLUXNET2015.
