# Supplementary material for: Assessing Spatial Representativeness of Global Flux Tower Eddy-Covariance Measurements Using Data from FLUXNET2015
Source: Sci Data. 2024 Jun 3;11:569. doi: 10.1038/s41597-024-03291-3 (PMC11148177; doi:10.1038/s41597-024-03291-3)
Supplement: Supplementary file 1 — Supplementary file for the standard deviation of the lateral wind speed prediction and uncertainties. [file 41597_2024_3291_MOESM1_ESM.docx]

**Supplementary Materials for**

**Assessing Spatial** **Representativeness of Global Flux Tower Eddy-Covariance Measurements Using Data from FLUXNET2015**

Junjun Fang^1,2^ , Jingchun Fang^1,2^, Baozhang Chen^1,2,3*^, Huifang Zhang^1,3^, Adil Dilawar^1,2^, Man Guo^1,4^, Shu’an Liu^5^

This PDF file includes:

Supplementary text S1, text S2

Fig S1-S3

Table S3, S4

References for SI reference citations

**Text S1. Random forest (RF) models for standard deviations of cross-wind velocity (sigma_v)**

We used a machine-learning algorithm (RF) to build models for predicting the standard deviations of cross-wind velocity (sigma_v) at sites where data is unavailable. RF constructs hierarchical binary decision trees grown from different bootstrap samples and randomly selected predicting variables^1^. The ranger package (a fast implementation of random forests) was used to carry out the model training and validation^2^. The workflow of the model training and validation are as follows.

For training the model, we used data from 37 sites (169 site-year) with available sigma_v (N = 1,332,045) at (half-) hours temporal resolution (details about sites please refer to Table S2 at https://doi.org/10.6084/m9.figshare.24866217)^3^. During the process of model development, a group k-fold cross-validation strategy (k = 5) was adopted to improve the prediction and fully evaluate the capacity of our model. The 5-fold cross-validation in our study could ensure that all data from one site will appear exactly in one fold which can validate the model capacity of extrapolation. Three metrics were used to evaluate the final model performance, including the root mean square error (RMSE), mean absolute error (MAE), and the coefficient of determination (R^2^). We could get 5 groups metrics with 5-fold cross-validation and the final result is the mean performance of all groups.

In the initial analyses, we chose 12 variables for their availability across research sites and theoretical relevance to cross-wind velocity (see the full list of variables in Figure S1c), including those associated with turbulence, atmospheric state, and measurements. A full model was trained using all 12 predicting variables. The validation result of the full model was shown in Figure S1a and Table S3, and the relative variable importance was calculated (Figure S1c). After that, the 7 most important predicting variables were identified, including friction velocity (USTAR), wind speed (WS), surface roughness length (Z0), incoming shortwave radiation (SW), measurement height above the zero-plane displacement height (HGT_M), canopy height (HGT_C), and atmospheric pressure (PA). Then, a reduced model was trained and validated using the identified 7 predicting variables. The results and variable importance were illustrated in Figure S1b, Figure S1d, and Table S3. We found that the reduced model showed robust performance (R^2^: 0.780, MESE:0.185 m s^-1^, MAE: 0.138 m s^-1^), nearly compatible with that from the full model. Thus, the reduced model was adopted and used to predict sigma_v for all sites with no available sigma_v data in the study.


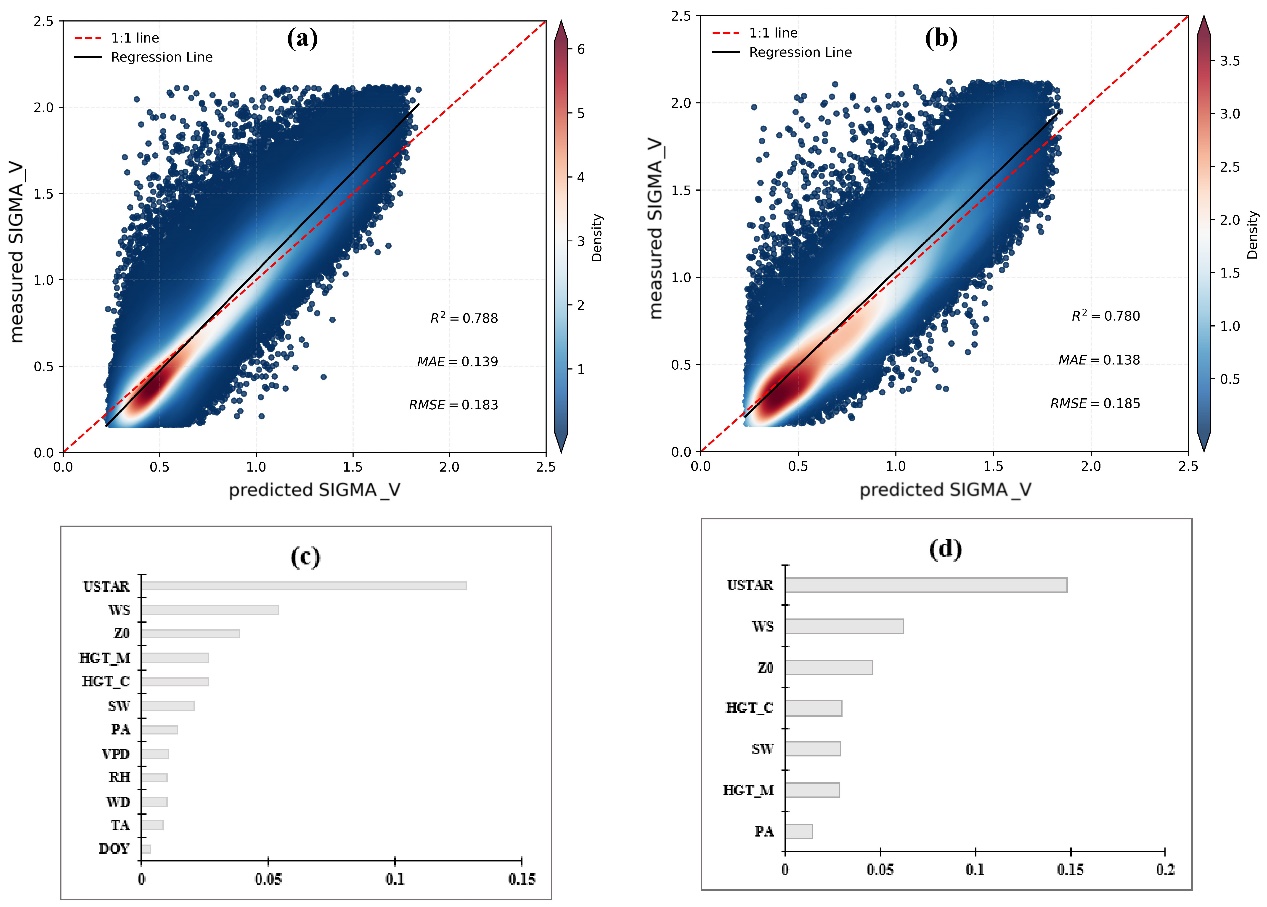


**Figure S1.** Description of the full random forests model and the reduced random forests model for prediction of the standard deviation of cross-wind velocity (sigma_v, m s^-1^). (a) is the full model performance during cross-validation, (b) is the reduced model performance during cross-validation, (c) is the variable importance of the predicting variables for the full model, and (d) is the variable importance of the predicting variables for the reduced model. The blue and dotted red lines show the linear regressions and the 1:1 reference line, the scatter color represents the amount of data. USTAR: friction velocity (m s^-1^), WS: wind speed (m s^-1^), z0: roughness length (m), SW: incoming short-wave radiation (W m^-2^), HGT_M: measurement height above the zero-plane displacement height (m), HGT_C: canopy height (m), VPD: vapor pressure deficit (hPa), WD: wind direction (decimal degree), TA: air temperature (°C), RH: relative humidity (%), PA: Atmospheric pressure (kpa), DOY: day of the year.

**Table S3.** Summary of the random forest models for predicting the standard deviation of cross-wind velocity (sigma_v, m s^-1^). RMSE: root mean square error. R^2^: Coefficient of determination. MAE: Mean absolute error.

| **Models** | **Statistics** | **Cross-validation values** |
| --- | --- | --- |
| Full model | RMSE | 0.183 |
|  | R^2^ | 0.788 |
|  | MAE | 0.139 |
| Reduced model | RMSE | 0.185 |
|  | R^2^ | 0.780 |
|  | MAE | 0.138 |

**Text S2. Uncertainty test of the sigma_v propagated to the annual cumulative footprint climatology (ACFC)**

We chose the ‘CA-TP1’ site (evergreen needleleaf forest) which with both measured and predicted sigma_v data for the uncertainty test to see how the sigma_v variable influences our Flux Footprint Prediction (FFP) model result. Because the RF reduced model with R^2^=0.780, so 22% uncertainty was added randomly to the sigma_v variable 5 times to drive the FFP model and analyze the differences between all the 7 results. We provided the annual cumulative footprint climatology contour (ACFCC) and footprint weights to directly compare the differences. We also provided the annual cumulative footprint climatology weighted (ACFCW) percentage of dominant land cover type and sensor location bias (SLB) between mean NDVI within 80% ACFCC and 80% ACFCW NDVI.

Figures S2 and S3 are the ACFCC and footprint weights from 7 scenarios of sigma_v. We can see that there is no big difference among the 7 scenarios, especially the measured and the predicted scenarios using the RF model. Table S4 is the statistical information about the 80% ACFCW percentage of dominant land cover type and SLB. The percentage of dominant land cover types using measured and predicted sigma_v are the same, and the difference among the 7 scenarios is 1.08%. From the perspective of SLB, ‘measured’ and ‘predicted’ scenarios have the same value owing to the same value of 80% ACFCW NDVI and mean NDCVI within 80% ACFCC, and the difference among 7 scenarios is 0.27% of SLB. Therefore, we conclude that although sigma_v is a necessary input parameter for the FFP model, it has little impact on the final ACFC result under the RF prediction with R^2^=0.78, and the impact can be a margin in the subsequent analysis of the percentage of dominant land cover type and SLB, which is consistent with the conclusion reached by Chu et al^4^.

**
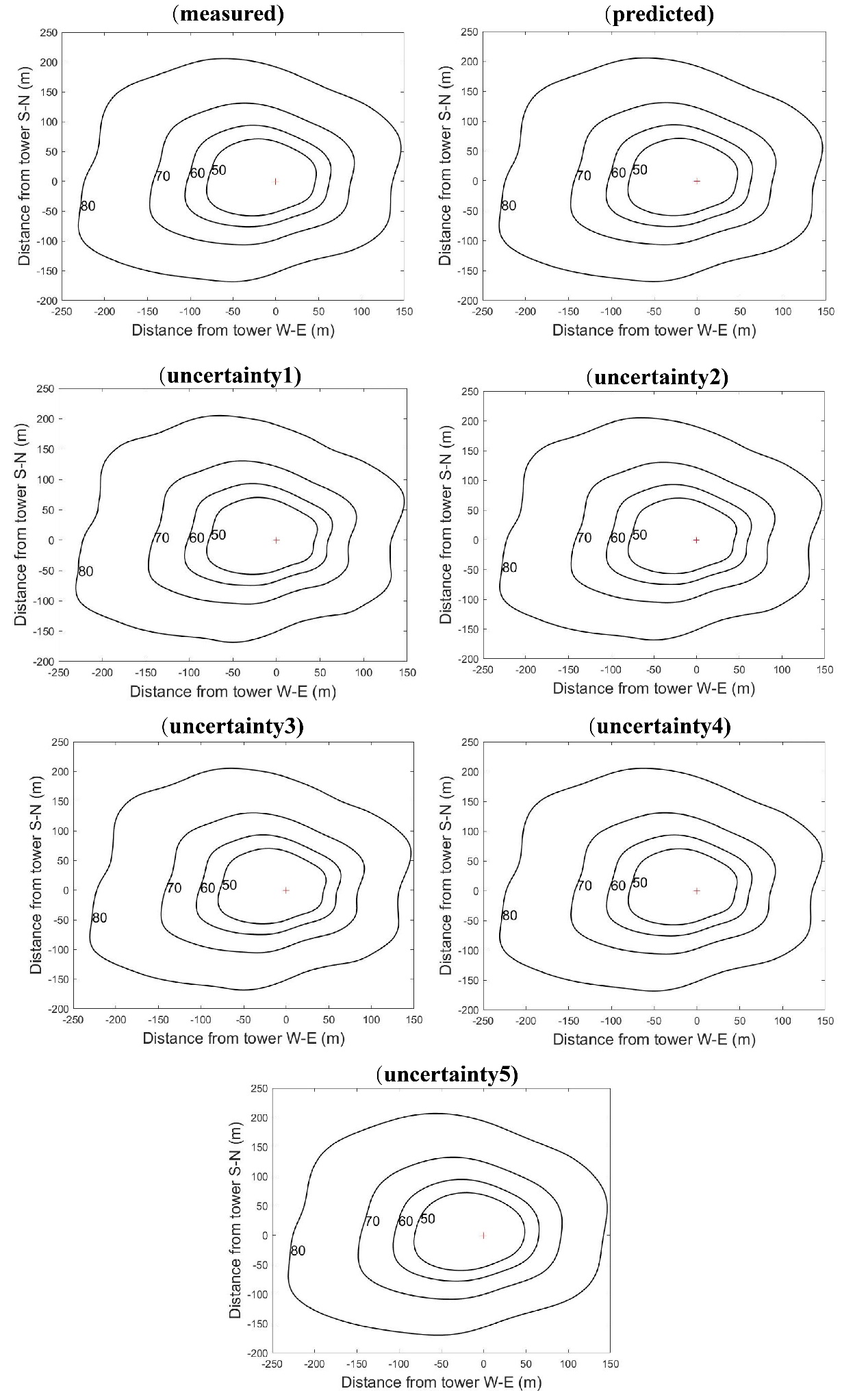
**

**Figure S2.** The annual cumulative footprint climatology contours (ACFCC) were calculated using the flux footprint prediction model (FFP) with 7 scenarios of sigma_v. The contours from inner to outer are 50%, 60%, 70%, and 80%, respectively. ‘measured’ denotes the FFP result using the measured sigma_v variable, ‘predicted’ represents the FFP result using predicted sigma_v variable with random forest, ‘uncertainty1’ to ‘uncertainty 5’ refer to the FFP result with 22% perturbation were added to measured sigma_v. The red cross symbol represents the location of this flux tower.

**
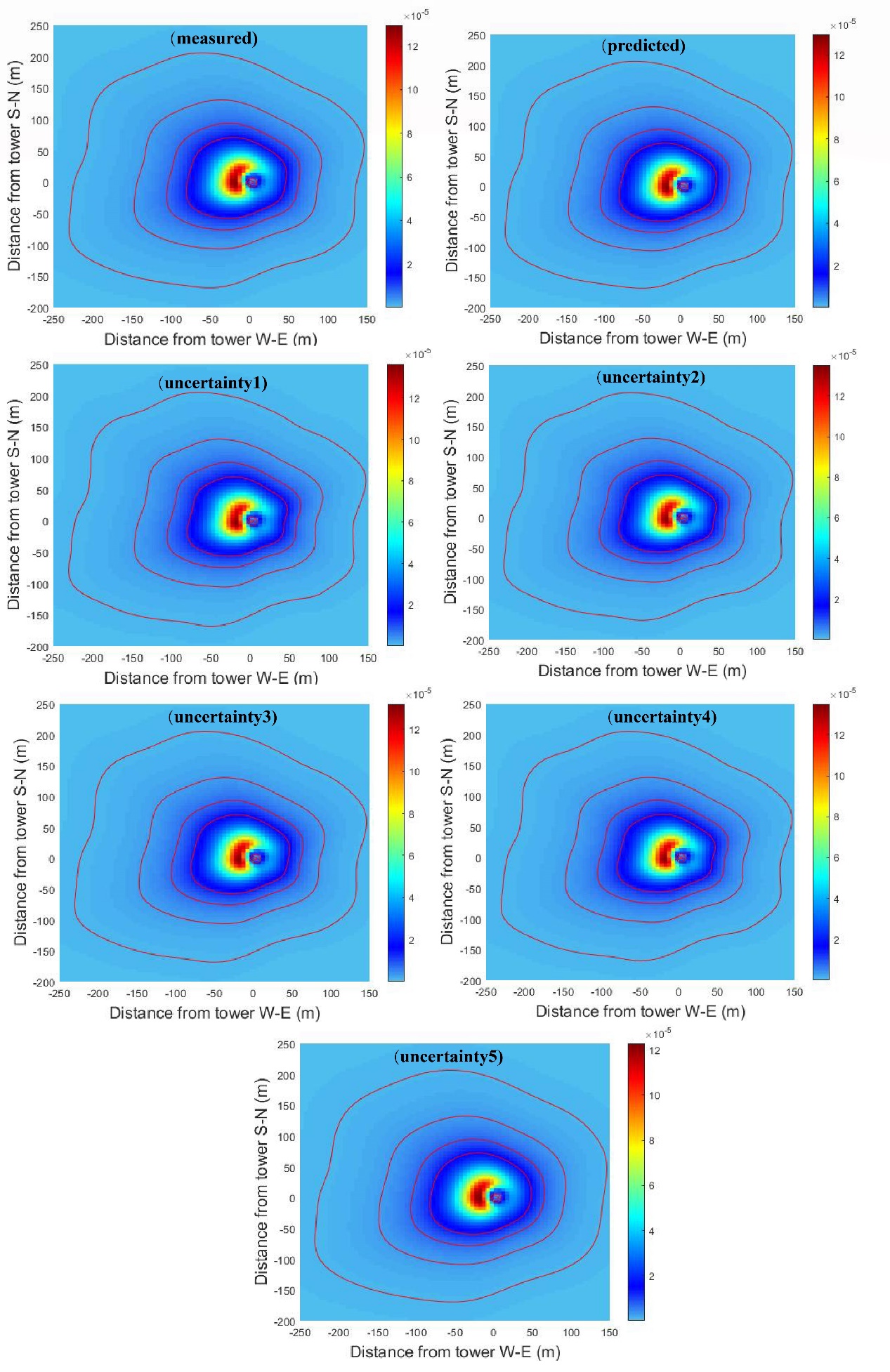
**

**Figure S3.** The spatial distribution of normalized annual cumulative footprint climatology weights (m^-2^) calculated using the flux footprint prediction model (FFP) with 7 scenarios of sigma_v. The contours from inner to outer are 50%, 60%, 70%, and 80%, respectively. ‘measured’ denotes the FFP result using the measured sigma_v variable, ‘predicted’ represents the FFP result using predicted sigma_v variable with random forest, ‘uncertainty1’ to ‘uncertainty 5’ refer to the FFP result with 22% perturbation were added to measured sigma_v.

**Table S4.** Statistical information about the footprint weighted percentage of dominant land cover type and sensor location bias in 7 different sigma_v scenarios. ‘measured’ denotes FFP result using measured sigma_v variable, ‘predicted’ represents FFP result using predicted sigma_v variable with random forest, ‘U1’ to ‘U5’ refers to the FFP result with 22% perturbation were added to measured sigma_v.

| Scenarios for sigma_v | Measured | Predicted | U1 | U2 | U3 | U4 | U5 |
| --- | --- | --- | --- | --- | --- | --- | --- |
| Percentage of dominant land cover type | 33.613 | 33.613 | 34.109 | 33.989 | 33.789 | 34.002 | 33.031 |
| 80% ACFCW NDVI | 0.730 | 0.730 | 0.731 | 0.731 | 0.730 | 0.731 | 0.730 |
| 80% ACFCC mean NDVI | 0.683 | 0.683 | 0.685 | 0.684 | 0.683 | 0.684 | 0.682 |
| Sensor location bais (%) | 6.931 | 6.931 | 6.736 | 6.775 | 6.951 | 6.775 | 7.001 |

[1] Breiman, L. Random forests. *Machine Learning*. **45,** 1, 5-32 (2001).

[2] Wright, M.N. and A. Ziegler. ranger: A Fast Implementation of Random Forests for High Dimensional Data in C plus plus and R. *Journal of Statistical Software*. **77,** 1, 1-17 (2017).

[3] Fang, J. Assessing Spatial Representativeness of Global Flux Tower Eddy-Covariance Measurements Using Data from FLUXNET2015. *Figshare,* [*https://doi.org/10.6084/m9.figshare.24884292*](https://doi.org/10.6084/m9.figshare.24884292). (2023).

[4] Chu, H., et al. Representativeness of Eddy-Covariance flux footprints for areas surrounding AmeriFlux sites. *Agricultural and Forest Meteorology*. **301,** (2021).
